# Use of *Periplaneta americana* as a Soybean Meal Substitute: A Step towards Sustainable Transformative Poultry Feeds

**DOI:** 10.3390/insects15090632

**Published:** 2024-08-23

**Authors:** Farwa Mustafa, Asif Sajjad, Roughaina Tahir, Mudssar Ali, Muhammad Sajjad, Asim Abbasi, Ehsaan Ullah Khan, Saba Zafar, Abeer Hashem, Graciela Dolores Avila-Quezada, Elsayed Fathi Abd_Allah

**Affiliations:** 1 Department of Entomology, Faculty of Agriculture and Environment, The Islamia University of Bahawalpur, Bahawalpur 63100, Pakistan; 2 Institute of Plant Protection, Muhammad Nawaz Shareef University of Agriculture Multan, Multan 60000, Pakistan; 3 Department of Entomology, Faculty of Agricultural Sciences and Technology, Bahauddin Zakariya University, Multan 60800, Pakistan; 4 Department of Entomology, University of Agriculture, Faisalabad 38040, Pakistan; asimuaf95@gmail.com; 5 Department of Animal Nutrition, Faculty of Animal Production and Technology, University of Veterinary and Animal Sciences, Lahore 54000, Pakistan; 6 Department of Biochemistry and Biotechnology, The Women University Multan, Multan 66000, Pakistan; 7 Botany and Microbiology Department, College of Science, King Saud University, P.O. Box 2460, Riyadh 11451, Saudi Arabia; 8 Facultad de Ciencias Agrotecnológicas, Universidad Autónoma de Chihuahua, Chihuahua 31350, Chihuahua, Mexico; 9 Plant Production Department, College of Food and Agricultural Sciences, King Saud University, P.O. Box 2460, Riyadh 11451, Saudi Arabia

**Keywords:** broiler, *H. illucens*, *P. americana*, meat quality, hematology and serum bio-chemistry

## Abstract

**Simple Summary:**

The global supply of the primary component of poultry feed, i.e., soybean meal, has dwindled due to an imbalance between demand and supply. Hence, there is an urgent need for exploring some cost-effective and environmentally friendly alternative protein sources to ensure sustainable poultry production. The outcomes of the present study exhibited that replacing 12% of soybean meal with either *Periplaneta americana* or *Hermetia illucens* improves the growth, blood hematology, gut morphometry, and meat quality of broilers. Consequently, these insects can serve as a viable alternative to traditional protein sources in poultry nutrition, offering a promising solution to meet the rising demand for animal protein, while mitigating the environmental impacts associated with conventional feed ingredients.

**Abstract:**

Insects are becoming increasingly popular as a sustainable and nutritious alternative protein source in poultry feeds, due to their high protein content, low environmental impact, and efficient feed conversion rates. Using insect-based feeds can reduce the need for traditional protein sources like soybean meal (SBM), which often contribute to environmental issues such as deforestation and high water consumption. The current experiments were devised to assess the impacts of the partial replacement of SBM with the American cockroach *Periplaneta americana* and black soldier fly *Hermetia illucens* on the performances, hematology, gut morphometry, and meat quality of male broilers (Ross 308). A total of 350, 1-day-old chicks weighing 40.05 ± 0.27 g were divided into 7 dietary treatments (5 pens/treatment and 10 birds/pen) at random, i.e., a 4, 8, or 12% SMB replacement with *P. americana* and *H. illucens*. Soybean meal was used as a basal diet and taken as a control. The results indicated that broilers fed on 12% *P. americana* or *H. illucens* showed significant improvements (*p* < 0.05) in feed conversion ratio, live weight, and daily weight gain. Hematological traits significantly improved (*p* < 0.05). A gut histology showed increased villus height, villus width, crypt depth, and villus height/crypt depth ratios, indicating improved nutrient absorption. Broiler meat fed on 12% *P. americana* meal had significantly higher redness and yellowness (*p* < 0.05). Substituting soybean meal with up to 12% *P. americana* or *H. illucens* meal in poultry feed can enhance the broilers’ growth performance, hematology, gut morphometry, and meat quality. Hence, these findings imply that *P. americana* or *H. illucens* meal are viable and constructive alternative protein sources in poultry nutrition, offering a sustainable approach to meet the increasing demand for animal protein across the world.

## 1. Introduction

The global demand for poultry meat and eggs is increasing due to the increasing human population, particularly in developing countries [1,2]. The cost effectiveness and sustainability of the poultry industry is mainly compromised due to the feed protein mainly obtained from soybean meal (SBM) and fish meal (FM) [3,4]. SBM is being extensively used in poultry feeds due to its well-suited profile of amino acids and high-quality protein content [5]. However, the rising prices of these protein sources make the poultry feed production chain less sustainable, especially in developing countries [6]. Moreover, SBM’s extensive use also brings up environmental concerns due to deforestation and higher water and pesticide usage [7,8]. Fish meal is also a high-quality protein source and used in poultry feed, but its quantity and accessibility rely heavily on the catch, which is projected to diminish in forthcoming years due to the depletion of marine resources [9,10]. Therefore, it was anticipated that the usage of insects in poultry feed as a viable alternative to traditional protein sources could benefit the poultry industry [3,11,12,13].

Nowadays, insects have attained growing interest as complementary protein sources in the poultry industry due to the high outlays for and constrained future availability of SBM and FM [14]. The promising amino acid profile, high chitin content, lauric acid, and antimicrobial agents in insects makes them a valuable source of proteins [15]. These bioactive compounds have been shown to have immunomodulatory, antibacterial, hypolipidemic, and growth-promoting effects [16,17]. Moreover, insect larvae have innate qualities that can fight off pathogens, reduce inflammation, and serve as antibiotics, helping to balance the microbial environment in the digestive tract of animals [18]. Insects are often considered as effective converters of bio-waste, do not need energy to maintain a high body temperature, and have excellent digestibility, which would otherwise lead to environmental problems [19,20]. Moreover, insects have the potential to reduce the load of bio-wastes by converting them into protein-rich sources in a limited space, efficiency in their feed conversion ratio, and high fecundity [21,22]. Furthermore, it is estimated that less than 1 ton of SBM can be produced per year from 1 hectare of land, while 150 tons of insect protein can be produced from the same area [23,24,25].

Several studies have explored the potential for substituting SBM with black soldier fly *Hermetia illucens* L. (Diptera: Stratiomyidae), yellow mealworm *Tenebrio molitor* L. (Coleoptera: Tenebrionidae), the house fly *Musca domestica* L. (Diptera: Bombycidae), and the silkworm moth *Bombyx mori* L. (Lepidoptera: Bombycidae) in poultry feeds [26,27,28]. Alterations in feed content and dietary protein source have been shown to affect broilers’ growth performance, hematology, and intestinal histomorphology [29,30,31]. These factors can have an impact on nutrient digestion, which in turn influences bird growth [32,33]. Insect meal can modify the microbial community of the birds, leading to the stimulation of humoral immunity, which in turn improves the growth performance and nutrient utilization of the hosts [34]. Similarly, the polysaccharides found in the exoskeleton of certain insects also improve the overall immunity of birds [35]. Intestinal functioning (nutritional digestion and absorption) is significantly influenced in its morphology including length, muscle thickness, villus height, crypt depth, and the ratio of villus height and crypt depth [36,37].

The daily feed intake is associated with the palatability of the diet, which in turn improves the growth performances of birds [38]. The growth performances and meat quality traits of broilers are significantly influenced by nutrition. The inclusion of *H. illucens* larvae in poultry feed has been shown to improve the growth performance, feed efficiency, and meat quality of the birds, owing to their highly digestible amino acids (AAs) and metabolizable energy content [39,40,41]. Meat color and texture are considered to be the most crucial factors of meat quality [42]. *H. illucens* and *M. domestica* based feeds have shown to impact the meat color of broilers [27,43,44].

The poultry sector primarily depends on SBM with a minor fraction of insect meal [45]. It is healthier and more balanced to switch from SBM to insects for poultry nutrition [46,47]. The exotic insect species of tropical areas (e.g., BSF) are being used in preparing animal and human diets [48]. There is also a need to explore native and invasive insect species that are suitable for mass multiplication, having a short life-cycle, high reproductive rate, and high nutritional value [49,50].

The American cockroach, *Periplaneta americana* (Blattodea: Blattidae) are bred in captivity, sold, and supplied as feed for livestock and to the humans as a food or for medicines (tonic) [51,52,53]. They are inexpensive to obtain and process and are readily accessible both on farms and households [54]. They are known for being a cost-effective protein source and suggested as alternative to the meat industry [55]. To strengthen the poultry industry, *P. americana* may be evaluated for the replacement of SBM in poultry feed. According to our knowledge, no information is available on using *P. americana* in poultry feed.

The objective of the present study was to assess the impact of substituting SBM (4, 8, and 12%) with *P. americana* on the growth performances, hematology, intestinal structure, and meat quality attributes of broilers. The exotic species of *H. illucens* was maintained at comparable replacement levels for comparison.

## 2. Materials and Methods

### 2.1. Insects

*Periplaneta americana* and *H. illucens* were reared on a combination of different bio-wastes, such as 50% poultry, 10% fruit, 10% vegetables, 10% grains, 10% household, and 10% fish, in the entomological research laboratory at the Department of Entomology, Faculty of Agriculture and Environment, The Islamia University of Bahawalpur, Pakistan. Fully grown larvae of *H. illucens* and adults of *P. americana* were separated from the leftover of their diets by sieving and washed in hot water to kill and eradicate the bio-wastes from their body. Then, these insects were dried for 24 h at 70 °C in a hot air oven and ground using a blender. The nutritional profile, energy content, and amino acid composition of *H. illucens* and *P. americana* were assessed using proximate analysis, bomb calorimetry, and Biochrome^®^ amino acid analyzer, as per the standard laboratory techniques at the Department of Animal Nutrition, UVAS, Ravi Campus Pattoki (Table 1).

### 2.2. Birds and Housing Conditions

The feeding trials were performed at the Avian Research and Training Centre, UVAS, Lahore. A total of 350 one-day-old chicks from the identical parental group were purchased from A.W. Breeders, Lahore, Pakistan. Over a span of 35 days, a total of five pens were assigned to each of the seven dietary treatments, with 10 birds in each pen. The seven experimental meals included increasing levels of SBM substitution, i.e., 4, 8, and 12%, with *P. americana*, and *H. illucens* (PA4, PA8, and PA12 for *P. americana*; HI4, HI8, and HI12 for *H. illucens*), while SBM was the basal diet considered as a control. The experiment periods had *ad libitum* feed and water available at all times. Proper ranking of the rice hull was maintained throughout the experiment. The temperature of the shed was kept at 34 °C for the first 5 days and then minimized to 26 °C till the end of the experiment. The chicks were immunized against infectious diseases at the hatchery. The mortality and health status of the broilers were visually monitored on a daily basis.

### 2.3. Formulation of Feeds

The meals were formulated based on the Ross [57] guidelines. The SBM was gradually replaced with *H. illucens* and *P. americana* at increasing levels of 4%, 8%, and 12% labeled as HI4, HI8, and HI12, PA4, PA8, and PA12, respectively. The meals were divided into three phases: starter (1–10 d), grower (11–24 d), and finisher (25–35 d). All the meals within each phase had the same caloric content and nitrogen level. The ingredients, nutrient composition, and energy content of the experimental treatments (starter, grower, and finisher) are presented in Table 2, Table 3 and Table 4.

### 2.4. Growth Performances

The growth performances of the broilers, such as live weight (LW), daily feed intake (DFI), average daily weight gain (ADG), and feed conversion ratio (FCR), were assessed to determine the effect on the meals, as described by Gariglio et al. [58]. The LW of the birds was recorded at the start of the experiment and at 10, 24, and 35 d of age. The DFI and ADG of the birds were estimated on an individual and pen basis, while the FCR was estimated for the growth intervals as well as for the entire duration of the trail.

### 2.5. Slaughtering

On 35 d, the birds (*n* = 10) were slaughtered following Sajjad et al. [59]. The LW was recorded prior to slaughter, and the head was removed after the bird was de-feathered and eviscerated. The *Pectoralis major* was deboned and chilled at 4 °C for 24 h after packaging in polyethylene bags, to assess meat quality traits following Ab Aziz et al. [60].

### 2.6. Hematology and Serum Bio-Chemistry

Hematological traits were assessed from two birds per pen (*n* = 10/treatment). At slaughtering, 2.5 mL blood was taken in serum-separating and EDTA tubes. For a complete blood count (CBC), blood smears were prepared from a blood droplet lacking anticoagulant [61]. The counts of white blood cells (leukocytes) and red blood cells (erythrocytes) were assessed, and the tubes lacking anticoagulant were allowed to clot upright at room temperature for two hours. Serum was separated by centrifugation at 700× *g* for 15 min. An automatic analyzer (Microlab 300 Semi-Automatic Chemistry Analyzer, Infitek Co., Ltd., Shandong, China) was used to check the glucose, creatinine, total protein (T. protein), globulin (Glob), cholesterol, albumin (Alb), and uric acid [62] levels. These tests were performed at the Diagnostic Lab of UVAS, Lahore.

### 2.7. Gut Histology

Small tissues (2 to 3 mm) of the jejunum and ileum were excised for intestinal morphometric indices, i.e., villus height (Vh), villus width (Vw), crypts depth (Cd), and Vh/Cd ratio. These segments were washed with 0.9% saline to remove feed content and preserved in 10% formalin solution for at least 72 h. Later on, the portions were placed onto slides, dyed using Lilee Meyer’s hematoxylin, and then counter-stained with eosin yellow. After staining, the oblique segments were examined under a light microscope (Meiji, Tokyo, Japan). ImageJ software (version 1.54j) was used for the analysis of images [63]. Measurements of Vh, Vw, Cd, and Vh/Cd were recorded from 20 well-oriented villi and crypts per bird [29,33]. The gut histological analysis was conducted at the Department of Anatomy and Histology, UVAS, Lahore.

### 2.8. Meat Quality

The impacts of *P. americana* and *H. illucens* meals on meat pH, shear force, meat color, and the drip and cooking losses of the meat were assessed using both the right and left *Pectoralis major*. These deboned breast muscles were chilled at 4 °C for 24 h. A pH meter (WTW, pH 3210 SET 2) was employed to evaluate the pH from three locations on the *Pectoralis major*. Two breast segments were utilized, one to estimate the drip loss and the other for cooking loss in percentage terms [64]. Subsequently, these cooked samples were chilled, reweighed to calculate the cooking. A Minolta CR-410 colorimete (Konica Minolta, Tokyo, Japan) was used in order to assess meat color parameters such as lightness (L*), redness (a*), and yellowness (b*) [65]. A scalpel handle blade was used to cut these breast samples in a direction parallel to the muscle fibers, resulting in rectangular pieces of approximately 1 h × 1 w × 2 L cm. A V-slot blade and a texture analyzer (TAXT plus texture analyzer, Godalming, UK) were utilized to assess the Warner–Bratzler shear force (measured in N/cm^2^).

### 2.9. Statistical Analysis

The pens were used as the experimental units to evaluate growth performance (*n* = 5), while individual birds were used for examining the hematology (*n* = 10), gut histology (*n* = 10), and meat quality parameters (*n* = 10). A completely randomized design was used for the data analysis, and Duncan’s multiple range test was conducted after a one-way analysis of variance ANOVA. Orthogonal polynomial contrasts were used to determine the linear and quadratic responses of *P. americana* and *H. illucens* to different meal concentrations. SPSS software (version 21 for Windows, SPSS Inc., Chicago, IL, USA) was used for the statistical analysis.

## 3. Results

### 3.1. Growth Performances

The live weight, average daily gain, feed intake, and feed conversion ratio are mentioned in Table 5. There was a significant difference (*p* < 0.05) between the dietary treatments in terms of the LW and ADG of the birds across the experimental periods, except the DOC (*p* > 0.05). At 10 d of age, the maximum LW was recorded in 12% *P. americana* and was statistically similar in all the remaining meals. The LW was maximum in 12% *H. illucens* and 12% *P. americana* meals and minimum in the control meal at 24 and 35 d of age. *H. illucens* meals led to a linear increase in LW at 10 d of age, while linear and quadratic responses were observed at 24 and 35 d of age. Similarly, *P. americana* meals revealed linear and quadratic effects on the LW of broilers.

The maximum ADG was registered in 12% *P. americana* during all the feeding periods except during d 25 and 35; it was maximum in 12% *H. illucens,* while it was the minimum in the control treatment. The orthogonal polynomial contrasts exhibited that the ADG increased both linearly and quadratically during d 1–10, d 11–24, and d 25–35, and linearly during d 1–35 among *H. illucens* meals. Similarly, there were linear and quadratic effects (*p* < 0.05) during the experimental periods among *P. americana* meals.

The FI did not show a significant difference (*p* > 0.05) between the meals. The FCR showed a significant difference (*p* < 0.05) between the dietary treatments across the feeding periods. During the ages of d 1–10 and d 25–35, the highest FCR was found in the control meal, while the lowest was in 12% *P. americana* meal. It was the highest in the control meal and the lowest in the 12% *H. illucens* and 12% *P. americana* meals during d 11–24 and d 1–35. Among the *H. illucens* meals, the FCR increased quadratically during d 1–10, and linearly during d 11–24 and d1–35, as well as responding linearly and quadratically during d 25–35. There were linear and quadratic responses during d 25–35 and d 1–35 and a linear response during d 11–24 among *P. americana* meals.

### 3.2. Hematology

#### Complete Blood Count (CBC)

The impacts of different meals on the hematological traits of the broilers are summarized in Table 6. The hematological traits differed significantly (*p* < 0.05) between the meals, except monocytes (*p* > 0.05). The highest hemoglobin, red blood cells, and mean corpuscular hemoglobin were recorded in 12% *P. americana,* while the minimum was in 4% *H. illucens*. The mean corpuscular hemoglobin concentration was maximum in 12% *H. illucens* and *P. americana*, while the minimum was in 4% *H. illucens*. HCT was maximum in 12% *H. illucens* and *P. americana,* while it was minimum in 8% *H. illucens*. The mean corpuscular volume was the maximum in 12% *H. illucens* and the minimum in 8% *H. illucens*. The platelets were the highest in 12% *P. americana* and the lowest in 8% *H. illucens*. The total leucocytes was maximum in 12% *H. illucens* and minimum in 4% *H. illucens,* while the total heterophils was maximum in 12% *P. americana* and minimum in 4% *P. americana*. There was a quadratic response in Hb and lymphocyte and a linear response in RBCs and MCHC to the *H. illucens* meals. There were linear and quadratic effects in HCT, MCV, MCH, platelets, TLC, and Heter to the *H. illucens* meals. Similarly, linear and quadratic responses were apparent in HCT, MCH, platelets, and lymphocytes to the *P. americana* meals, while there were quadratic effect in MCV, MCHC, TLC, and heterophils to the *P. americana* meals.

### 3.3. Serum Bio-Chemistry

The serum biochemistry traits are mentioned in Table 6. Serum bio-chemistry traits showed a significant difference (*p* < 0.05). The highest creatinine was recorded in the control meal and the lowest in the 12% *H. illucens* and *P. americana* diets. Glucose and cholesterol levels were the highest in the control and the lowest in 12% *H. illucens*. Uric acid was the maximum in the control and the minimum in 12% *P. americana*. The highest total protein, albumin, and globulin was in 12% *P. americana* and the lowest in the control meal.

Creatinine, cholesterol, total protein, and globulin registered a quadratic response in the polynomial contrasts, while albumin and uric acid showed a linear response among *H. illucens* meals. The *H. illucens* meals showed linear and quadratic responses to glucose level. Similarly, glucose and globulin exhibited linear and quadratic responses to the *P. americana* diets. Cholesterol, albumin, and uric acid showed a linear response, while total protein displayed a quadratic response among *P. americana* meals.

### 3.4. Gut Histology

The effects of the meals, the sites of the small intestine, and their interactions on the gut morphometric indices of the broilers are mentioned in Table 7. There was a significant difference (*p* < 0.001) in the experimental meals, the intestinal sites, and their interactions on gut morphometric indices such as Vh, Cd, Vw and Vh/Cd. There was an interaction between the meals and the intestinal sites, with the highest and comparable Vh values recorded at the jejunum of the broilers fed the control, 4, 8, and 12% *H. illucens* and 4% *P. Americana*—the highest at the jejunum and ileum sites when fed meals containing 8 and 12% *P. americana* and the lowest at the ileum site when fed the control, 4, 8, and 12% *H. illucens*. The largest Cd value was found at the jejunum site of the broiler fed with control meal, while the smallest Cd values were measured at the jejunum and ileum sites on 12% *P. americana* meal. The highest and comparable Vw values were recorded at the jejunum using the 12% *H. illucens* and *P. americana* meals. The lowest and comparable Vw values were noticed when the broilers were fed the control meal at the jejunum and ileum sites: 4 and 8% *H. illucens* at the ileum site and 8% *P. americana* at the jejunum. The largest and comparable Vh/Cd values were recorded at the jejunum when the broilers were fed with 8% *H. illucens*, 12% *H. illucens,* and 4% *P. americana* meals and at the jejunum and ileum sites when fed 8 and 12% *P. americana* meals, while the lowest and comparable were at the ileum when fed on the control, 4, 8, and 12% *H. illucens* and 4% *P. americana* meals.

### 3.5. Meat Quality

The effects of different meals on the meat quality parameters of the broilers are described in Table 8. The cooking loss, L*, a*, and b* differed significantly (*p* < 0.05) among the experimental meals, while the meat pH, drip loss, and shear force did not show a significant difference (*p* > 0.05). The highest cooking loss and L* was noticed in the control, while the lowest was in the 12% *P. americana* and 12% *H. illucens* meals. The a* was the highest and statistically similar in 8% *H. illucens*, 12% *H. illucens*, 8% *P. americana,* and 12% *P. americana* and the lowest in the control meal. The maximum b* was recorded in 12% *P. americana* and 12% *H. illucens* and the minimum in all the remaining meals. The polynomial contrasts revealed both linear and quadratic effects in cooking loss, a*, and b*, while there was a quadratic response in L* to the *H. illucens* meals. Similarly, cooking loss had linear and quadratic effects, whereas L*, a*, and b* had a linear effect among *P. americana* meals.

## 4. Discussion

Natural insect food sources have traditionally been an excellent choice for poultry and wild birds [66]. They are used as a protein source to meet the nutritional requirements of poultry, reduce production costs without compromising quality, and increase production rates [12]. The present research aimed to assess the effect of replacing SBM with invasive *P. americana*—in comparison with the commercially used *H. illucens*—on the growth performances, hematology, gut morphology, and meat quality traits of male broilers.

### 4.1. Growth Performances

Our outcomes showed that the maximum LW and ADG were recorded in 12% *P. americana* and 12% *H. illucens* meals. Feed ingredients, particularly protein and essential amino acids, are critical for optimal feed utilization, which significantly impacts bird growth performance [67,68]. The digestibility of the dietary protein promotes body weight gain by accumulating protein content due to nutrient changes and feed energy content [69,70].

The results of the current study are aligned with Loponte [41] and Dabbou et al. [71], who reported that replacing 10% and 50% SBM with *H. illucens* improved the LW and ADG in Ross 308 broilers and barbary partridges, respectively. Hwangbo et al. [72] and Ballitoc and Sun [28] reported that the body weight increased in Ross broilers fed on 10% *M. domestica* and 10% *T. molitor* meals, respectively. Biasato et al. [73] and Ramos-Elorduy et al. [74] suggested that substituting 5% to 10% SBM with *T. molitor* had no effect on growth performances in Arbor Acres and Hubbard hybrid broilers. Biasato et al. [75], Biasato et al. [76], and Vasilopoulos et al. [77] suggested that replacing 10% to 15% SBM with *T. molitor* significantly increased LW and ADG in the fast-growing Ross 708 and Ross 308 and the intermediate-growing Hubbard hybrid.

In this study, the FI did not differ among the experimental meals. Feed intake increases as the feed’s protein and lipid content decrease, to compensate for the protein deficiency needed for proper growth [78,79,80]. Similar results were obtained by Chu et al. [81], Hwangbo et al. [72] and Ramos-Elorduy et al. [74] when broilers were fed on 3 to 9% *H. illucens*, 5% to 20% *M. domestica,* and 5% to 10% *T. molitor* meals, respectively. Gariglio et al. [58] also observed similar findings in Muscovy duck when fed on 9% *H. illucens.*

The FCR improved in broilers in our study when fed on 12% *H. illucens* and 12% *P. americana* meals. The FCR serves as an essential indicator of feed conversion efficiency in livestock production, which is mainly influenced by the nutritional value of the meal [82,83]. Substituting conventional protein sources with insects can enhance the FCR due to high nutrients, sufficient amino acids (e.g., lauric acid), an appealing palatability, easy digestion, energy efficiency, and reduced antinutritional factors [84,85]. This leads to improved growth rates and a more efficient conversion of feed into body mass in broilers [86]. Our outcomes are in line with Kim et al. [87] and Lalev et al. [88], who noticed an improved FCR at a 5% and 10% substitution of SBM with *H. illucens* and *Bombyx mori* in broilers. In broilers, an 8% inclusion of *M. domestica* and 10% to 30% inclusion of *T. molitor* have been reported to improve the FCR [17,89,90].

### 4.2. Hematology

Our results revealed that meals containing 12% *P. americana* and 12% *H. illucens* had the maximum Hb, RBCs, MCH, MCHC, HCT, MCV, platelets, lymphocytes, and hematocrit. Blood production in the bone marrow is directly influenced by diet and the welfare of the birds [91,92]. The feed’s protein content depends on the variability in the hematological and biochemical characteristics of broilers [93,94]. Incorporating insect protein in poultry feed boosts RBC production and strengthens the immune system [95]. Biasato et al. [75] reported that the 10% *T. molitor* diet increased the RBCs in female Ross 708 broilers. Vilela et al. [96] observed that blood lymphocytes increased at 20% *H. illucens* in broilers. Our results align with Schiavone et al. [97], who found that substituting 50% to 100% soybean oil with *H. illucens* did not affect the blood traits in broilers (Ross 500). This discrepancy might be attributed to the variation in feed composition and breed of the boiler, i.e., soybean oil has high fat, while SBM has a high protein and fiber content.

### 4.3. Serum Bio-Chemistry

In the present study, 12% *P. americana* and 12% *H. illucens* meals showed the maximum levels of total protein, globulin, and albumin, while they had the minimum cholesterol, glucose, creatinine, and uric acid. The chelating effects of chitin and chitosan may reduce blood uric acid, glucose, and cholesterol levels [16,98,99,100]. The birds’ metabolic function improved as cholesterol, glucose, creatinine, and uric acid levels decreased in their blood [101,102]. The nutritional composition of the diet may strengthen birds’ immunity, increasing their resilience to diseases and infections [97]. According to Bovera et al. [103], the levels of total proteins and globulin are typically affected by the isoenergetic and isoprotenic compositions of dietary treatments. Our results are consonant with prior studies by Bovera et al. [17] and Sedgh-Gooya et al. [104], who stated that 5% to 100% *T. molitor* diets increased the total protein and globulin levels while decreasing the uric acid in Shaver brown broilers and Bovans white laying hens. Some other studies by Schiavone et al. [97], Kim et al. [87], Schiavone et al. [105], and Sypniewski et al. [101] revealed that the levels of creatinine, uric acid, and glucose did not differ by substituting 5% to 100% soybean oil with *H. illucens* in broilers and turkey birds.

### 4.4. Gut Histology

In the present study, the interactions between the experimental diets and the sites of the small intestine were significant. The highest Vh value was observed in the jejunum of the broilers fed the control, 4%, 8%, and 12% *H. illucens* and 4% *P. americana* and at the jejunum and ileum sites fed the 8% and 12% *P. americana* meals. The highest Vw and Vh/Cd value were registered at the jejunum of the broilers fed the 12% *H. illucens* and *P. americana* meals, with the Cd being the smallest. Insects contain chitin, antioxidants, and antimicrobial peptides that play a key role in the development of the animal gut [66,106]. Morphological changes in the gut epithelium such as Vh and Cd are induced by the nutritive value of the diets [107]. The efficient digestion and assimilation of nutrients, as well as overall animal health, depend on the surface area of absorptive epithelium in the small intestine [36,108]. The absorption of nutrients rises as the Vh increases, while reduced nutrient uptake is indicative of poor gut development due to shorter villi [109]. Our findings are consistence with Sajjad et al. [59], who reported a significant interaction between the dietary treatments and intestinal sites (jejunum and ileum) of broilers when fed with 12% *H. illucens* and 12% *S. frugiperda*. The results of the current study diverged from the earlier studies conducted by Biasato et al. [73] and Biasato et al. [110], who found no changes in the gut histological indices such as Vh, Vw, Cd, and Vh/Cd of Ross 708 and Hubbard hybrid broilers fed with 7.5% and 15% *T. molitor* meals. Dabbou et al. [71] and Dabbou et al. [111] also observed similar trends in Ross 308 fed with 15% defatted *H. illucens* meal and modified fatted *H. illucens* meal. The distinctions in results between our study and the previous research could be attributed to variations in breeds, the nutritional profile of various insect species and their developmental stages, and the experimental conditions.

### 4.5. Meat Quality

Our results revealed that 12% *P. americana* and 12% *H. illucens* meals had the lowest cooking loss and L*, while having the highest a* and b*. Meat color and texture are crucial factors that influence consumers’ acceptance of meat and its products [112,113]. The mono- and polyunsaturated fatty acid composition—oleic and linoleic—of the diets cause variations in the meat color [114]. Moreover, 20% *H. illucens* meal has high concentrations of saturated fatty acids; i.e., lauric acid, myristic acid, and eicosapentaenoic acid improved the meat color in broilers [96]. Several factors can influence this effect, including the pigments of the feed raw materials and the other constituents of the formulation. Consumer perception of the color may depend on their preferences [115]. The rich protein content of the diet can affect the water-holding capacity of breast meat [116]. Leiber et al. [117] reported that replacing 7.8% SBM with *H. illucens* decreased cooking losses in Ross 308 broilers. Our results are analogous with the findings of Murawska et al. [118], Kim et al. [119], and Schiavone et al. [44], who found that growing the substitution levels of SBM with *H. illucens* reduced the cooking loss while increasing the b* in broilers (Ross 308). The meat color among bird species is affected by the nature of their diet e.g., a* increased in broilers [44,96] while decreasing in quail [27] when fed with 15% *H. illucens*. Pieterse et al. [43] reported that the water-holding capacity of meat muscles increased and thawing and cooking losses decreased in broilers (Ross 308) fed with 10% *M. domestica*. Nonetheless, various factors such as diet, age, genetics, stress, and slaughtering methods can influence the quality of the meat [112].

## 5. Conclusions

The present study provides innovative insights into the effects of replacing SBM with the invasive *P. americana* in poultry feed on the growth performance, hematology, gut histology, and meat quality traits of the broilers. The results were statistically in consonance with commercially used *H. illucens*. The meal containing 12% *P. americana* and 12% *H. illucens* resulted in the optimal growth performance, hematology, gut histology, and meat quality traits of the chickens. The efficient feed intake and feed conversion ratio underscore the potential of *P. americana* as a sustainable alternative for poultry growth. Variations in the meat physicochemical indices provide valuable information about modifications in dietary treatments that may have an impact on consumer acceptability. Future research should investigate the optimal inclusion levels of other insect species in the feed of various poultry breeds, as well as the impact of insect-based diets on the gut microbiomes of poultry birds.

## Figures and Tables

**Table 1:** Nutritional profile, energy content, and amino acid composition of *H. illucens* and *P. Americana.*

Nutrient Composition (%)	*H. illucens*	*P. americana*
Dry matter (as such basis)	91.00	92.00
Crude protein	42.00	40.40
Crude fat	32.60	11.02
Ash	10.70	6.78
Crude fiber	9.40	6.08
Nitrogen-free extract	5.30	29.72
Calcium	2.10	1.29
Phosphorus (available)	0.94	0.52
Energy Content (kcal/kg)	*H. illucens*	*P. americana*
Gross energy	5010	4250
Metabolizable energy ^a^	1464	1239
Essential amino acids (%)	*H. illucens*	*P. americana*
Arginine	*2.26*	5.36
Lysine	3.13	8.20
Methionine	1.22	2.37
Threonine	1.88	3.53
Leucine	3.11	7.86
Isoleucine	2.54	4.02
Valine	3.08	4.21
Dispensable Amino Acids (%)	*H. illucens*	*P. americana*
Cysteine	0.40	3.65
Tryptophan	0.27	1.66
Glycine	3.06	3.43
Glutamic acid	11.32	12.07
Proline	2.89	2.50
Tyrosine	3.31	3.31
Phenylalanine	4.32	3.47

^a^ Metabolizable energy was estimated using the equations of Abdullahi et al. [56]. The sample was analyzed in triplicate.

**Table 2:** Ingredients, nutrient composition, and energy content of meals.

	Starter Meals						
Ingredients (%)	Control	HI4	HI8	HI12	PA4	PA8	PA12
Corn grain	51.17	51.93	54.35	55.57	51.92	54.10	54.80
Wheat bran	4.00	4.00	3.00	3.00	4.00	3.00	3.00
Rice polishing	4.00	4.00	4.00	4.00	4.00	4.00	3.00
Soybean oil	4.00	3.00	1.80	0.80	3.00	2.00	2.40
Soybean meal	28.50	25.00	21.00	17.00	25.00	21.00	17.00
Fish meal	6.00	6.00	6.00	6.00	6.00	6.00	6.00
HI meals	-	4.00	8.00	12.00	-	-	-
PA meals	-	-	-	-	4.00	8.00	12.00
L-Lysine HCl	0.03	-	-	-	-	-	-
DL-Methionine	0.15	0.12	0.10	0.07	0.12	0.10	0.07
Common salt	0.30	0.30	0.30	0.30	0.30	0.30	0.30
Limestone	1.75	1.55	1.35	1.16	1.55	1.50	1.40
Micro Min Premix ^a^	0.05	0.05	0.05	0.05	0.05	0.05	0.05
Vitamin Premix ^a^	0.05	0.05	0.05	0.05	0.05	0.05	0.05
Total	100	100	100	100	100	100	100
Nutrient composition, %	Control	HI4	HI8	HI12	PA4	PA8	PA12
Dry matter	89.40	89.40	89.50	89.40	89.40	89.50	89.40
Crude protein	23.00	23.03	23.00	22.98	23.03	23.00	23.02
Ether extract	6.65	6.72	6.80	6.86	6.62	6.70	6.76
Crude fiber	4.06	4.22	4.27	4.36	4.22	4.27	4.26
Ash	3.62	3.85	3.96	3.80	3.85	3.96	3.80
Nitrogen-free extract (NFE) ^b^	61.00	60.98	60.84	60.94	60.98	60.84	60.94
Calcium	0.95	0.95	0.95	0.95	0.95	0.95	0.95
Phosphorus (available) ^c^	0.51	0.51	0.52	0.52	0.51	0.52	0.52
Lysine	1.32	1.32	1.33	1.33	1.32	1.33	1.33
Methionine	0.55	0.55	0.55	0.55	0.55	0.55	0.55
Threonine	0.88	0.89	0.90	0.90	0.89	0.90	0.90
Valine	1.03	1.04	1.03	1.04	1.04	1.03	1.04
Arginine	1.41	1.41	1.42	1.40	1.41	1.42	1.40
Leucine	1.46	1.46	1.44	1.45	1.46	1.44	1.45
Isoleucine	0.88	0.89	0.88	0.89	0.89	0.88	0.89
Energy content (kcal/kg)	Control	HI4	HI8	HI12	PA4	PA8	PA12
Gross Energy ^d^	4606	4593	4598	4604	4593	4598	4604
Metabolizable Energy ^e^	2980	2978	2973	2974	2978	2973	2974

HI4, 4% *H. illucens*; HI8, 8% *H. illucens*; HI12, 12% *H. illucens*; PA4, 4% *P. americana*; PA8, 8% *P. americana*; PA12, 12% *P. americana*; ^a^ standard vitamin and mineral premix of Trouw Nutrition GB, The Netherlands, was used for the starter meals; ^b^ NFE, ^c^ phosphorus (avail.) and ^e^ metabolizable energy were calculated, while all other ingredients were analyzed. ^d^ Gross energy was estimated using the Bomb Calorimeter IKA C2000 (Werke GmbH, Sauerlach, Germany); ^e^ ME was estimated using the equations of Abdullahi et al. [56].

**Table 3:** Ingredients, nutrient composition, and energy content of meals.

	Grower Meals						
Ingredients (%)	Control	HI4	HI8	HI12	PA4	PA8	PA12
Corn grain	51.17	51.93	54.35	55.57	55.00	56.10	57.20
Wheat bran	4.00	4.00	3.00	3.00	5.00	4.00	3.00
Rice polishing	4.00	4.00	4.00	4.00	4.00	4.00	4.00
Soybean oil	4.00	3.00	1.80	0.80	3.30	3.00	2.80
Soybean meal	28.50	25.00	21.00	17.00	22.00	18.30	14.50
Fish meal	6.00	6.00	6.00	6.00	5.00	5.00	5.00
HI meals	-	4.00	8.00	12.00	-	-	-
PA meals	-	-	-	-	4.00	8.00	12.00
L-Lysine HCl	0.03	-	-	-	-	-	-
DL-Methionine	0.15	0.12	0.10	0.07	0.05	-	-
Common salt	0.30	0.30	0.30	0.30	0.30	0.30	0.30
Limestone	1.75	1.55	1.35	1.16	1.25	1.20	1.10
Micro Min Premix ^a^	0.05	0.05	0.05	0.05	0.05	0.05	0.05
Vitamin Premix ^a^	0.05	0.05	0.05	0.05	0.05	0.05	0.05
Total	100	100	100	100	100	100	100
Nutrient composition, %	Control	HI4	HI8	HI12	PA4	PA8	PA12
Dry matter	89.40	89.40	89.50	89.40	89.30	89.50	89.40
Crude protein	23.00	23.03	23.00	22.98	21.50	21.51	21.50
Ether extract	6.65	6.72	6.80	6.86	7.08	7.10	7.09
Crude fiber	4.06	4.22	4.27	4.36	4.18	4.27	4.31
Ash	3.62	3.85	3.96	3.80	3.55	3.68	3.81
Nitrogen-free extract (NFE) ^b^	61.00	60.98	60.84	60.94	62.20	61.89	61.13
Calcium	0.95	0.95	0.95	0.95	0.75	0.75	0.75
Phosphorus (available) ^c^	0.51	0.51	0.52	0.52	0.42	0.42	0.43
Lysine	1.32	1.32	1.33	1.33	1.19	1.19	1.19
Methionine	0.55	0.55	0.55	0.55	0.51	0.51	0.51
Threonine	0.88	0.89	0.90	0.90	0.79	0.79	0.79
Valine	1.03	1.04	1.03	1.04	0.92	0.91	0.92
Arginine	1.41	1.41	1.42	1.40	1.27	1.28	1.27
Leucine	1.46	1.46	1.44	1.45	1.30	1.31	1.30
Isoleucine	0.88	0.89	0.88	0.89	0.81	0.80	0.80
Energy content (kcal/kg)	Control	HI4	HI8	HI12	PA4	PA8	PA12
Gross Energy ^d^	4606	4593	4598	4604	4659	4664	4671
Metabolizable Energy ^e^	2980	2978	2973	2974	3049	3048	3051

HI4, 4% *H. illucens*; HI8, 8% *H. illucens*; HI12, 12% *H. illucens*; PA4, 4% *P. americana*; PA8, 8% *P. americana*; PA12, 12% *P. americana*; ^a^ standard vitamin and mineral premix of Trouw Nutrition GB, The Netherlands, was used for the grower meals; ^b^ NFE, ^c^ phosphorus (avail.) and ^e^ metabolizable energy were calculated, while all other ingredients were analyzed. ^d^ Gross energy was estimated using the Bomb Calorimeter IKA C2000 (Werke GmbH, Germany); ^e^ ME was estimated using the equations of Abdullahi et al. [56].

**Table 4:** Ingredients, nutrient composition, and energy content of meals.

	Finisher Meals						
Ingredients (%)	Control	HI4	HI8	HI12	PA4	PA8	PA12
Corn grain	57.27	58.65	61.00	63.24	58.17	59.47	60.85
Wheat bran	5.00	5.00	4.00	3.00	5.00	4.00	3.00
Rice polishing	4.00	4.00	4.00	4.00	4.00	4.00	4.00
Soybean oil	5.50	4.30	3.20	2.00	4.80	4.40	4.10
Soybean meal	22.50	18.50	14.50	10.70	18.50	14.50	10.70
Fish meal	4.00	4.00	4.00	4.00	4.00	4.00	4.00
HI meals	-	4.00	8.00	12.00	-	-	-
PA meals	-	-	-	-	4.00	8.00	12.00
L-Lysine HCl	0.04	0.03	0.03	-	-	-	-
DL-Methionine	0.14	0.12	0.09	0.06	0.08	0.03	-
Common salt	0.30	0.30	0.30	0.30	0.30	0.30	0.30
Limestone	1.15	1.00	0.78	0.60	1.05	1.00	0.95
Micro Min Premix ^a^	0.05	0.05	0.05	0.05	0.05	0.05	0.05
Vitamin Premix ^a^	0.05	0.05	0.05	0.05	0.05	0.05	0.05
Total	100	100	100	100	100	100	100
Nutrient composition, %	Control	HI4	HI8	HI12	PA4	PA8	PA12
Dry matter	89.40	89.30	89.60	89.50	89.30	89.60	89.50
Crude protein	19.52	19.53	19.51	19.52	19.53	29.51	19.52
Ether extract	8.06	8.08	8.11	8.13	8.08	8.11	8.10
Crude fiber	3.99	4.04	4.08	4.10	4.04	4.08	4.10
Ash	3.44	3.54	3.53	3.57	3.54	3.53	3.57
NFE ^b^	63.82	63.69	63.50	63.40	63.69	63.50	63.43
Calcium	0.65	0.66	0.65	0.66	0.66	0.65	0.66
Phosphorus (available) ^c^	0.36	0.36	0.36	0.36	0.36	0.36	0.36
Lysine	1.08	1.08	1.09	1.08	1.08	1.09	1.08
Methionine	0.48	0.48	0.48	0.48	0.48	0.48	0.48
Threonine	0.72	0.72	0.72	0.72	0.72	0.72	0.72
Valine	0.84	0.84	0.84	0.85	0.84	0.84	0.85
Arginine	1.17	1.18	1.17	1.18	1.18	1.17	1.18
Leucine	1.19	1.19	1.19	1.20	1.19	1.19	1.20
Isoleucine	0.75	0.75	0.76	0.75	0.75	0.76	0.75
Energy content (kcal/kg)	Control	HI4	HI8	HI12	PA4	PA8	PA12
Gross Energy ^d^	4733	4728	4719	4713	4728	4719	4713
Metabolizable Energy ^e^	3124	3112	3105	3102	3112	3105	3102

HI4, 4% *H. illucens*; HI8, 8% *H. illucens*; HI12, 12% *H. illucens*; PA4, 4% *P. americana*; PA8, 8% *P. americana*; PA12, 12% *P. americana*; ^a^ standard vitamin and mineral premix of Trouw Nutrition GB, The Netherlands, was used for the finisher meals; ^b^ NFE, ^c^ phosphorus (avail.) and ^e^ metabolizable energy were calculated, while all other ingredients were analyzed. ^d^ Gross energy was estimated using the Bomb Calorimeter IKA C2000 (Werke GmbH, Germany); ^e^ ME was estimated using the equations of Abdullahi et al. [56].

**Table 5:** Growth performances of broilers affected by *H. illucens* and *P. americana* meals.

Items	Control	*Hermetia illucens*			*Periplaneta americana*			SEM	*p*-Value				
		HI4	HI8	HI12	PA4	PA8	PA12		ANOVA	HI Lin.	HI Quad.	PA Lin.	PA Quad.
Live weight, g													
DOC	40.18	40.06	39.95	40.06	40.02	40.03	40.08	0.06	0.99	0.83	0.51	0.634	0.752
10 d	247.3 ^bc^	257.5 ^abc^	236.39 ^c^	306.17 ^ab^	265.67 ^abc^	294.52 ^abc^	318.83 ^a^	3.47	0.04	0.01	0.943	<0.001	<0.001
24 d	1022.67 ^f^	1076.5 ^e^	1133.33 ^c^	1216.67 a	1111.5 ^d^	1172.83 ^b^	1205.67 ^a^	14.58	<0.001	<0.001	<0.001	<0.001	<0.001
35 d	1990.3 ^d^	2012.27 ^cd^	2120.8 ^b^	2126.3 ^ab^	2038.3 ^c^	2105.4 ^b^	2159.67 ^a^	14.05	<0.001	<0.001	0.009	<0.001	<0.001
Daily weight gain, g													
1–10 d	22.98 ^f^	24.72 ^e^	26.58 ^d^	30.62 ^b^	25.76 ^ab^	29.44 ^c^	31.88 ^a^	0.66	<0.001	<0.001	<0.001	<0.001	<0.001
11–24 d	55.38 ^f^	58.50 ^e^	63.38 ^bc^	64.07 ^ab^	60.41 ^d^	62.75 ^c^	65.05 ^a^	0.72	<0.001	<0.001	0.025	<0.001	0.046
25–35 d	82.66 ^e^	85.07 ^d^	86.73 ^c^	89.78 ^a^	84.26 ^d^	84.78 ^d^	87.96 ^b^	0.51	<0.001	<0.001	<0.001	<0.001	<0.001
1–35 d	56.87 ^d^	57.49 ^cd^	60.59 ^b^	60.76 ^ab^	58.24 ^c^	60.15 ^b^	61.71 ^a^	0.4	<0.001	<0.001	0.601	<0.001	<0.001
Feed intake, g													
1–10 d	22.74	23.84	23.18	23.59	24.36	23.84	23.11	0.42	0.98	0.660	0.720	0.655	0.506
11–24 d	94.31	92.78	92.17	90.15	91.23	90.84	90.66	0.64	0.7	0.074	0.868	0.178	0.923
25–35 d	177.44	177.19	176.40	176.77	177.76	176.01	178.03	0.37	0.82	0.561	0.774	0.923	0.770
1–35 d	98.16	97.94	97.25	96.84	97.79	96.90	97.27	0.3	0.91	0.329	0.928	0.367	0.839
Feed conversion ratio, g/g													
1–10 d	0.98 ^a^	0.93 ^ab^	0.92 ^ab^	0.77 ^bc^	0.92 ^ab^	0.81 ^abc^	0.72 ^c^	0.03	0.03	0.082	0.041	0.081	0.079
11–24 d	1.70 ^a^	1.59 ^b^	1.44 ^cd^	1.39 ^d^	1.51 ^bc^	1.45 ^cd^	1.43 ^cd^	0.05	<0.001	<0.001	0.139	<0.001	0.713
25–35 d	2.14 ^a^	2.09 ^bc^	2.05 ^cd^	2.02 ^d^	2.11 ^ab^	2.08 ^bc^	1.96 ^e^	0.01	<0.001	<0.001	<0.001	0.044	0.029
1–35 d	1.73 ^a^	1.70 ^ab^	1.60 ^c^	1.59 ^c^	1.68 ^b^	1.61 ^c^	1.58 ^c^	0.01	<0.001	<0.001	0.593	<0.001	0.004

Orthogonal polynomial contrast; HI lin., *H. illucens* linear; HI quad., *H. illucens* quadratic; PA lin., *P. americana* linear; PA quad., *P. americana*. The values marked by different letters in superscript within the rows were significantly different (*p* < 0.05).

**Table 6:** Hematology and serum bio-chemistry of broilers affected by *H. illucens* and *P. americana* meals.

Traits	Control	*Hermetia illucens*			*Periplaneta americana*			SEM	*p*-Value				
		HI4	HI8	HI12	PA4	PA8	PA12		ANOVA	HI Lin.	HI Quad.	PA Lin.	PA Quad.
Hematology													
Hb	8.9 ^c^	8.33 ^d^	9.9 ^ab^	10 ^ab^	9.67 ^b^	9.7 ^b^	10.3 ^a^	0.15	<0.001	0.12	0.001	0.592	0.946
RBCs	2.83 ^bc^	2.57 ^c^	3.03 ^bc^	3.1 ^ab^	2.87 ^bc^	3.03 ^bc^	3.53 ^a^	0.08	0.02	0.03	0.561	0.175	0.348
HCT	32.36 ^c^	30.07 ^e^	26.63 ^f^	35.3 ^a^	31.06 ^d^	33.2 ^b^	35.33 ^a^	0.64	<0.001	<0.001	<0.001	<0.001	<0.001
MCV	98.06 ^d^	89.73 ^e^	83.17 ^f^	113.83 ^a^	100.13 ^c^	97.23 ^d^	105.33 ^b^	2.06	<0.001	0.004	<0.001	0.981	<0.001
MCH	31.77 ^c^	25.73 ^f^	29.5 ^e^	32.87 ^b^	31.23 ^d^	29.37 ^e^	34.23 ^a^	0.58	<0.001	<0.001	<0.001	<0.001	<0.001
MCHC	32.43 d	30.3 ^e^	33.33 ^c^	36.27 ^a^	32.73 ^d^	34.27 ^b^	36.53 ^a^	0.46	<0.001	<0.001	0.154	0.221	0.004
Platelets	13,966.67 ^e^	16,666.17 ^d^	13,033.23 ^f^	29,033.13 ^b^	14,666.47 ^e^	23,033.33 ^c^	31,033.30 ^a^	1552.16	<0.001	<0.001	<0.001	<0.001	<0.001
TLC	11,000 ^d^	6533.33 ^g^	10,066.67 ^e^	17,466.67 ^a^	9200 ^f^	13,100 ^c^	14,033.33 ^b^	744.15	<0.001	0.004	<0.001	0.585	<0.001
Heter.	36.07 ^d^	44.33 ^c^	50.33 ^b^	50.33 ^b^	36.67 ^d^	50.33 ^b^	62.67 ^a^	1.92	<0.001	<0.001	<0.001	0.539	<0.001
Lym.	33.67 ^c^	44.67 ^b^	45 ^b^	56.67 ^a^	33.33 ^c^	45.33 ^b^	56.33 ^a^	1.99	<0.001	0.09	<0.001	<0.001	0.005
Mono.	2	2	2	2	2	2	2	0.14	0.45	0.429	0.105	0.734	0.855
Serum bio-chemistry													
Creatinine	0.53 ^a^	0.43 ^ab^	0.4 ^b^	0.23 ^c^	0.33 ^bc^	0.33 ^bc^	0.23 ^c^	0.03	<0.001	0.156	0.04	0.08	0.394
Glucose	212.67 ^a^	201.33 ^b^	196.33 ^c^	135.67 ^f^	204.67 ^b^	172.33 ^e^	192.33 ^d^	1.45	<0.001	<0.001	<0.001	<0.001	<0.001
Cholesterol	152.67 ^a^	154.33 ^a^	142.17 ^bc^	120.33 ^d^	140.33 ^bc^	143.33 ^b^	138.67 ^c^	1.2	<0.001	0.173	0.007	<0.001	0.224
T. Protein	2.73 ^d^	3.33 ^bc^	3.37 ^bc^	3.53 ^b^	2.93 ^d^	3.23 ^c^	4.13 ^a^	0.1	<0.001	0.565	0.005	0.857	<0.001
Alb.	1.2 ^d^	1.3 ^cd^	1.4 ^bc^	1.5 ^ab^	1.3 ^cd^	1.5 ^ab^	1.6 ^a^	0.03	0.03	0.01	0.58	0.002	0.487
Glob.	1.43 ^e^	1.63 ^de^	2.17 ^bc^	2.33 ^ab^	1.63 ^de^	1.9 ^cd^	2.63 ^a^	0.01	<0.001	0.07	0.006	0.02	<0.001
Uric acid	4.63 ^a^	3.43 ^d^	4.13 ^b^	3.63 ^cd^	4.23 ^b^	3.83 ^c^	3.53 ^d^	0.02	<0.001	<0.001	0.63	<0.001	0.832

Traits as follow: Hb g/dL, hemoglobin; RBCs × 10^6^/µL, red blood cells; HCT %, hematocrits; MCV fL, mean corpuscular volume; MCH pg, mean corpuscular hemoglobin; MCHC, mean corpuscular hemoglobin concentration; platelets/µL; TLC × 10^3^/µL, total leucocytes; Heter. (%), heterophils; Lym. (%), lymphocytes; Mono. (%), monocytes; creatinine mg/dL; glucose mg/dL; cholesterol mg/dL; T. protein g/dL, total protein; Alb. g/dL, albumin; Glob. g/dL, globulin; uric acid g/dL. The values marked by different letters in superscript within the rows were significantly different (*p* < 0.05).

**Table 7:** Gut morphometric indices of broilers as affected by *H. illucens* and *P. americana* meals.

Diet	Site	Vh	Cd	Vw	Vh/Cd
Control	Jejunum	1407.2 ^abcd^	187.76 ^a^	43.40 ^ef^	7.92 ^bcdef^
	Ileum	1026.6 ^e^	177.82 ^cd^	43.20 ^ef^	5.47 ^g^
HI4	Jejunum	1357.2 ^abcd^	182.28 ^b^	61.67 ^bc^	7.65 ^bcdef^
	Ileum	1133.0 ^de^	177.53 ^cd^	46.01 ^def^	6.21 ^fg^
HI8	Jejunum	1421.4 ^abcd^	175.62 ^de^	74.70 ^a^	8.26 ^abcd^
	Ileum	1106.9 ^de^	172.03 ^fg^	47.53 ^def^	6.31 ^efg^
HI12	Jejunum	1559.7 ^ab^	164.26 ^h^	70.16 ^ab^	9.38 ^ab^
	Ileum	1158.5 ^cde^	166.28 ^h^	51.05 ^cde^	7.04 ^cdefg^
PA4	Jejunum	1473.2 ^abc^	178.84 ^c^	50.63 ^cde^	8.41 ^abcd^
	Ileum	1237.1 ^bcde^	175.07 ^de^	49.09 ^def^	6.92 ^defg^
PA8	Jejunum	1392.4 ^abcd^	173.18 ^ef^	37.91 ^f^	8.22 ^abcde^
	Ileum	1541.7 ^ab^	169.45 ^g^	50.79 ^cde^	8.90 ^abc^
PA12	Jejunum	1602.7 ^a^	161.23 ^i^	74.79 ^a^	9.95 ^a^
	Ileum	1478.7 ^abc^	161.00 ^i^	55.43 ^cd^	9.17 ^ab^
PSEM		18.29	3.40	1.69	0.34
Probability					
Meals		<0.001	<0.001	<0.001	<0.001
Sites		<0.001	<0.001	<0.001	<0.001
Meals * Sites		0.002	<0.001	0.003	<0.001

PSEM, pooled standard error of mean. * Effects of two variables have been combined in ANOVA analyses. The values marked by different letters in superscript within the rows were significantly different (*p* < 0.05).

**Table 8:** Meat quality traits of broilers affected by *H. illucens* and *P. americana* meals.

Traits	Control	*Hermetia illucens*			*Periplaneta americana*			SEM	*p*-Value				
		HI4	HI8	HI12	PA4	PA8	PA12		ANOVA	HI Lin. ^1^	HI Quad. ^1^	PA Lin. ^1^	PA Quad. ^1^
Meat pH	6.09	6.11	6.12	6.14	6.13	6.11	6.12	0.01	0.282	0.566	0.491	0.189	0.625
Drip loss (%)	2.45	2.32	2.45	2.19	2.30	2.11	2.42	0.81	0.693	0.427	0.507	0.304	0.985
Shear force	61.03	61.02	60.84	61.07	60.86	60.88	60.93	0.03	0.159	0.805	0.120	0.219	0.435
Cooking loss (%)	33.32 ^a^	26.12 ^c^	18.10 ^e^	18.70 ^e^	29.32 ^b^	21.10 ^d^	20.20 ^de^	1.68	<0.001	<0.001	<0.001	<0.001	<0.001
L*	58.75 ^a^	57.17 ^ab^	54.70 ^bc^	48.94 ^d^	56.67 ^abc^	53.14 ^c^	49.61 ^d^	0.86	<0.001	0.447	0.001	0.004	0.701
a*	12.35 ^c^	13.87 ^b^	16.02 ^a^	16.29 ^a^	13.70 ^b^	16.13 ^a^	16.23 ^a^	0.25	<0.001	0.04	<0.001	<0.001	0.351
b*	19.15 ^b^	18.44 ^b^	18.31 ^b^	21.32 ^a^	19.28 ^b^	19.09 ^b^	21.03 ^a^	0.6	0.03	0.026	<0.001	<0.001	0.868

^1^ Orthogonal polynomial contrast, HI lin., *H. illucens* linear; HI quad., *H. illucens* quadratic; PA lin., *P. americana* linear; PA quad., *P. americana*; L*, lightness; a*, redness; b*, yellowness; The values marked by different letters in superscript within the rows were significantly different (*p* < 0.05).

## Data Availability

The data are contained within the article. Further inquiries can be directed to corresponding authors.
